# What makes them stick? A genetic analysis of biofilm formation of an infective endocarditis-causing *Streptococcus canis* strain using transposon directed insertion-site sequencing

**DOI:** 10.3389/fcimb.2026.1777632

**Published:** 2026-04-30

**Authors:** Miriam Katsburg, Anna Kopenhagen, Mathias Müsken, Inga Eichhorn, Silver A. Wolf, Simone Bergmann, Marcus Fulde

**Affiliations:** 1 Institute of Microbiology and Epizootics, Centre for Infection Medicine, Freie Universität Berlin, Berlin, Germany; 2 Veterinary Centre for Resistance Research (TZR), Freie Universität Berlin, Berlin, Germany; 3 Institute of Microbiology, Technische Universität Braunschweig, Braunschweig, Germany; 4 Central Facility for Microscopy, Helmholtz Centre for Infection Research, Braunschweig, Germany; 5 Genome Competence Centre (MF1), Robert Koch Institute (RKI), Berlin, Germany

**Keywords:** biofilm, extracellular polysaccharides, infective endocarditis, quorum sensing, streptococcal adhesion factor, TraDIS

## Abstract

**Background:**

*Streptococcus canis* is an emerging zoonotic pathogen capable of causing infective endocarditis (IE) in animals and humans. In IE, bacterial biofilms form vegetations on heart valves, protecting microbes from antibiotics and immune responses, which complicates treatment and promotes chronic infection.

**Methods:**

To identify genes required for biofilm development, we performed transposon-directed insertion site sequencing (TraDIS) in combination with a biofilm formation assay on a fibrin matrix under physiologically relevant flow conditions. Mutant libraries were screened for deficiencies in biofilm formation, followed by pathway enrichment and targeted functional assays.

**Results:**

Mutants impaired in biofilm formation were enriched for disruptions in carbohydrate metabolism, cell wall biogenesis, and quorum sensing pathways. The *rfb* operon and *galE*, genes essential for extracellular polysaccharide synthesis, were identified as key contributors. Pathway analysis highlighted quorum-sensing and HIF-1 signaling as regulators of metabolic adaptation and matrix production under flow. Biofilm formation by the IE strain was inhibited by carvacrol, an inhibitor of LuxS-dependent quorum sensing. Deletion of the fibronectin-binding serum opacity factor (ScSOF) significantly reduced biofilm formation on fibronectin-coated surfaces and altered matrix composition, demonstrating its role in host matrix–dependent adhesion.

**Discussion:**

These findings provide the first genome-wide characterization of biofilm-associated gene networks in *S. canis*, revealing how metabolic pathways, quorum sensing, and host adhesion factors interact to promote endocardial biofilm formation.

## 1 Introduction

Infective endocarditis (IE) is a life-threatening condition characterized by the colonization of heart valves or endocardial surfaces by microorganisms, resulting in the formation of vegetations composed of bacteria, fibrin, platelets, and extracellular matrix material (Widmer et al., 2006; Witten et al., 2024). A major factor limiting successful treatment of IE is biofilm formation, which confers bacteria with increased tolerance to antibiotics and host immune defenses. This is a huge concern in veterinary medicine, as medical intervention is often the only feasible treatment method (Reagan et al., 2022; Katsburg et al., 2023). Within these structured communities of vegetations, bacteria embed themselves within a self-produced matrix composed of extracellular polysaccharides, proteins, and extracellular DNA (eDNA), leading to chronic and relapsing infections that are difficult to eradicate even with aggressive therapy (Lerche et al., 2021b).

*Streptococcus canis*, a group G β-hemolytic streptococcus primarily associated with dogs and cats (Sykes et al., 2006; Lysková et al., 2007), has increasingly been recognized as an opportunistic zoonotic pathogen capable of causing severe infections in humans, including bacteremia, necrotizing fasciitis, and infective endocarditis (Amsallem et al., 2014; Lacave et al., 2016). Although rare in humans, *S. canis* endocarditis cases in dogs highlight the pathogens capacity to form vegetative lesions typical of biofilm-driven infections (Reagan et al., 2022). Due to close interactions with pets, this organism can also impact human health (Mališová et al., 2019; Wang et al., 2024). Despite this clinical relevance, the molecular basis of biofilm formation in *S. canis* remains largely unexplored (Fukushima et al., 2022). Most knowledge of streptococcal biofilms is derived from species such as *S. pyogenes*, *S. agalactiae*, and *S. mutans*, where biofilm development involves multiple stages: initial adhesion, microcolony formation, maturation, and dispersal, all of which are regulated by a network of surface proteins, secreted factors, and quorum-sensing systems (Nobbs et al., 2009; Yadav et al., 2020).

In streptococci, quorum sensing (QS), including the LuxS/autoinducer-2 (AI-2) system, plays a pivotal role in coordinating population-wide behaviors such as competence, virulence, and biofilm maturation. QS enables bacteria to sense cell density and collectively regulate the expression of genes required for extracellular matrix production and structural organization. Disruption of these signaling pathways frequently leads to attenuated biofilm formation, suggesting that *S. canis* may similarly rely on density-dependent regulation for establishing infection on cardiac tissue.

Biofilm formation in streptococci is further influenced by surface-anchored adhesins and virulence factors, which mediate attachment to host tissues and interbacterial aggregation. Proteins such as the M protein, fibronectin-binding proteins, and serum opacity factor (SOF) have been shown to facilitate adhesion and immune evasion in *S. pyogenes* (Oehmcke et al., 2004, Oehmcke et al., 2010; Alamiri et al., 2020). *S. canis* encodes homologues of several of these proteins, including SOF, which is known to interact with high-density lipoproteins (HDL) and fibronectin in other streptococci (Courtney et al., 1999; Katerov et al., 2000; Baums et al., 2006; Timmer et al., 2006; Courtney et al., 2009). However, the contribution of SOF to biofilm formation and host interaction in *S. canis* has not been elucidated.

In this study, we employed Transposon-Directed Insertion Site Sequencing (TraDIS) to identify genetic determinants of biofilm formation in *S. canis*. By comparing the non-biofilm forming transposon mutant population with the input library, we define key genes involved in adhesion, extracellular matrix production, and quorum sensing. Understanding the molecular mechanisms that govern *S. canis* biofilm formation in the context of IE will provide crucial insights into its pathogenesis and reveal potential targets for therapeutic intervention.

## 2 Materials and methods

### 2.1 Bacterial strains and growth conditions

*Streptococcus canis* strains IMT49926 (Katsburg et al., 2023), G361 (Fulde et al., 2011), 49926Δ*sof* (Katsburg et al., 2025) were used as well as a ISS1 transposon library made in IMT49926 as described previously (Katsburg et al., 2025). Bacteria were cultured on Columbia agar plates with 5% sheep blood (BD) or in Todd Hewitt broth (THB) (Roth) unless indicated otherwise.

### 2.2 Biofilm formation under flow conditions

A fibrin matrix was formed on an Ibidi µ-Slide I Luer 3D^®^ by incubating 10 mg/mL of plasminogen-depleted human fibrinogen (Millipore) in PBS with 1.0 KU/mL thrombin (from bovine plasma; MP Biomedicals) in each well at 37 °C overnight. A colony of the *S. canis* transposon library was grown overnight in THY (THB + 1.5% yeast extract). The culture was washed once with PBS and resuspended to OD_600_ of 0.05 in Brain-Heart-Infusion-Broth (Roth) with 2% (w/v) glucose (BHIgluc) added. This suspension was added to the fibrin matrix and the Ibidi^®^ system was filled with BHIgluc. The Ibidi 3D Slide^®^ was connected to a software- controlled pneumatic pump (Ibidi, Germany) to simulate physiological shear stress conditions (10 dyn/cm^2^). Biofilms were allowed to grow for 24 hours before harvesting non-adherent bacteria by washing the matrix with PBS. The non-adherent bacteria were grown overnight at 37 °C.

### 2.3 DNA extraction

Bacterial cells were harvested by centrifugation and frozen or directly resuspended in 180 μL enzyme solution (20 mg/mL lysozyme, 30 mM Tris-Cl pH 8, 2 mM EDTA, 10% Triton X-100) for 1 h at 37 °C. Subsequently, 20 μL proteinase K and 1 μL RNase A (5 μg/μL) were added, followed by 200 μL AL buffer (QIAmp DNA Mini Kit, Qiagen) and incubation at 56 °C for 1 h. After adding 200 μL ethanol, the mixture was applied to a spin column for DNA purification, and the protocol of the kit was followed from here. DNA was eluted with 30 μL Milli-Q water and quantified spectrophotometrically. This protocol enables efficient isolation of high-quality genomic DNA from *S. canis* for further molecular analyses.

### 2.4 Scanning electron microscopy

For scanning electron microscopy (SEM), the Ibidi^®^ slides were washed twice with 1ml of PBS after biofilm formation. Samples were fixed in an SEM fixation solution containing 720 µL of TE buffer, 200 µL of paraformaldehyde (25%) and 8 µL of glutaraldehyde (25%). SEM sample preparation and imaging were performed at the Central Facility for Microscopy (ZEIM) at the Helmholtz Center for Infection Research as described before (Kopenhagen et al., 2022).

### 2.5 Static biofilm formation on glass and fibrinogen-coated surfaces

A 24-well plate with 12 mm glass coverslips was either left uncoated or coated with human fibronectin (50 µg/mL, Corning). After washing with PBS, a bacterial culture suspension of OD_600_ 0.05 in BHIgluc was added to the wells as before. The outer wells of the plate were not used due to the possible impact of evaporation. After 72 hours, biofilms were washed three times with PBS and fixed overnight at 4 °C with 4% paraformaldehyde in PBS. For staining, following reagents were used: DAPI, wheat germ agglutin AF488, FilmTracer™ SYPRO™ Ruby Biofilm matrix stain and ProLong Diamond antifade mountant (all Invitrogen). Images were acquired as z-stacks every 0.5 µm using a DMI6000B fluorescence microscope equipped with a 63x/1.40 oil immersion objective and operated with LAS X software (Leica, Germany). Mean fluorescence intensity was quantified for every fluorescent channel independently without summations by ImageJ using the RAW image files. Acquisition settings were kept constant across all samples for a given channel. Fluorescence values used for MFI quantification were not saturated.

### 2.6 DNA preparation and sequencing

DNA was extracted from transposon libraries after biofilm formation under flow conditions and prepared and sequenced as previously described (Katsburg et al., 2025).

### 2.7 TraDIS analysis

*For generation of the transposon library, S. canis* IMT49926 was transformed with plasmid pGh9:IS*S1* by electroporation and a transposon library was generated as described previously [26]. The library comprised 21879 unique insertion sites across the genome. Of 1914 annotated genes, 1727 (90.2%) contained at least one transposon insertion with an average of 9.7 insertions per gene and a median of 6 insertions per gene. A total of 187 genes showed no insertions.

TraDIS analysis was performed as described prior (Katsburg et al., 2025). Briefly, genomic sequences from the *S. canis* library used for infection analyses are named “input pool” and sequences from bacteria recovered after biofilm formation assays are named “output pool”. From input pool and from all output pools, raw fastq files were analysed using the Bio-TraDIS (v1.4.5) scripts of the Sanger Welcome Trust Institute (https://github.com/sanger-pathogens/Bio-Tradis) in a similar way as Charbonneau et al (Barquist et al., 2016; Charbonneau et al., 2017). First, contigs were trimmed to 68 bp using the Trimmomatic read trimming tool (v0.40) (Bolger et al., 2014). Next, the bacteria_tradis pipeline script filtered reads according to the specified transposon tag (GAGAAAACTTTGCAACAGAACC). After tag removal, the remaining 46 bp of *S. canis* DNA were mapped to the IMT49926 reference genome (NCBI Bioproject PRNJA945807) using SMALT short read mapper (v0.7.6)(http://www.sanger.ac.uk/resources/software/smalt/), producing a plot file of insertion sites for downstream analysis. A transposon tag mismatch of 1 was allowed, while the mapping threshold was set to 100%. The tradis_comparison.R script was used to compare the input (control) and output pools (conditions) using the tradis_gene_insert_sites output files from each experimental replicate (n=3) of the pools. This generates a volcano plot illustrating log 2-fold change (log2FC) and -log2 (q-values) for every mutagenized gene as well as a csv file summarizing this information. The csv files were used for further downstream analysis.

The IMT49926 genome was functionally annotated to obtain the proteome using the eggNOG-mapper 2.1.12 (Cantalapiedra et al., 2021). COG annotations were subsequently added to the csv output files from the TraDIS analysis. To visualize the data, the csv file was analysed with R (v4.4.0). A subset was generated of genes with log2FC> 2, q value <0.05 and COG G, M, V and T which encode carbohydrate transport and metabolism, cell wall biogenesis, defense mechanisms and signal transduction mechanisms, which are clusters of genes that we expected to potentially be involved in biofilm formation. A heatmap of this subset was created with pheatmap (v1.0.12). The q value of 0.05 instead of 0.01 was chosen to show the major cell wall component SCM-2, which encodes the *S. canis* M-protein type 2 (Lapschies et al., 2023).

### 2.8 Pathway analysis

The proteome of IMT49926 was uploaded to STRING (v12.0) (https://version-12-0.string-db.org/organism/STRG0A12MZE) (Szklarczyk et al., 2023). From the comparative analysis of the output pool, all genes with log2FC>2, q value<0.01 and a predicted function were extracted and utilized for pathway analysis. The STRING network was loaded in Cytoscape (v3.10.4) (Shannon et al., 2003) and the ClueGO app (v2.5.10) (Bindea et al., 2009) was used to visualize involved KEGG pathways.

### 2.9 Carvacrol treatment of biofilms

Carvacrol (Sigma-Aldrich) was dissolved in dimethyl sulfoxide (DMSO) to create a stock solution of 100 mg/mL. Using a crystal violet assay as described by Wijesundara et al (Wijesundara et al., 2022), the minimal biofilm inhibitory concentration (MBIC) was determined for both IMT49926 and G361. To determine the effect of carvacrol on biofilms, the static biofilm formation assay on a glass surface was used as described before. Carvacrol in the indicated concentrations was added before incubation or after 24 hours for the preformed biofilms. As a negative control, DMSO was added to the cultures in the same concentration as the highest carvacrol treatment. Staining and imaging were then executed as described for the static biofilm formation assay. Mean fluorescence intensity was quantified by ImageJ for every fluorescent channel independently without summations using the RAW image files. Acquisition settings were kept constant across all samples for a given channel. Fluorescence values used for MFI quantification were not saturated.

### 2.10 Statistical analysis

Statistical analysis was performed by one-way ANOVA with *post hoc* comparisons using the Prism software (v8.0) (GraphPad) with which graphs were generated.

## 3 Results

### 3.1 Identification of genes involved in biofilm formation under flow conditions on a fibrin matrix

**Figure 1:**
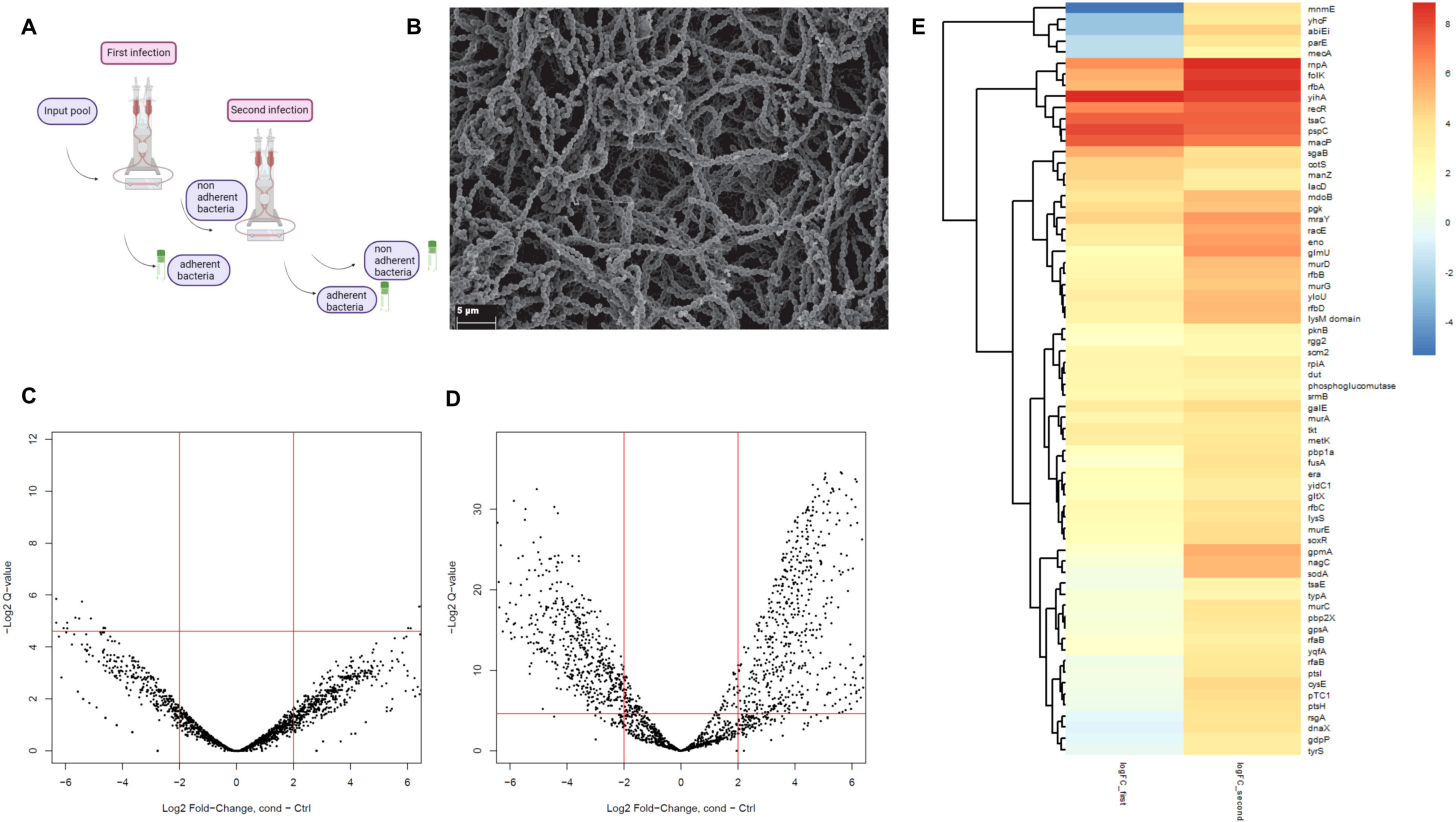
**(A)** Schematic overview of TraDIS output pool generation under flow conditions. **(B)** Representative SEM visualization of a 24h-old biofilm of (S) canis IMT49926 formed on a fibrin matrix. **(C)** Volcano plot of a comparative analysis of the generated output pool (non-adherent bacteria after first selection step) and the input library showing the log2FC of all genes after infection selection compared to the input pool as control. **(D)** Volcano plot of a comparative analysis of the generated output pool (non-adherent bacteria after second selection step) and the input library showing the log2FC of all genes after infection selection compared to the input pool as control. **(E)** Heatmap of genes of COGs G, M, V and T which encode carbohydrate transport and metabolism, cell wall biogenesis, defense mechanisms and signal transduction mechanisms with the log2FC values after first selection in the first column and after second selection in the second column. Only genes with log2FC>2 and q<0.05 after the second selection step are included.

To identify genes that play a role in biofilm formation, a random ISS1 transposon library that was generated in our lab previously from the clinical endocarditis *S. canis* strain IMT49926 was used combined with a biofilm formation assay under flow conditions on a fibrin matrix, as depicted in **Figure 1A**. This assay enables us to more closely replicate the physiological conditions of biofilm formation in infective endocarditis. The screen for bacterial factors mediating biofilm development was achieved by a negative selection process, which filters bacterial transposon mutants with functional loss in biofilm formation. The loss in biofilm formation leads to a loss of biofilm-mediated bacterial adherence to fibrin surfaces due to single transposon insertions. The constructed biofilms were imaged by electron microscopy to confirm that a biofilm had formed on the fibrin matrix (**Figure 1B**). To separate the non-adherent transposon mutants from the adherent mutants, two rounds of infection of the fibrin matrix were performed as seen in the schematic overview (**Figure 1A**). The iterative selection process resulted in significantly enriched genes and also detects genes with moderate phenotypic contributions. Afterwards, both bacteria attached to the fibrin matrix and non-attached (non-adherent) bacteria circulating in the supernatant were separately harvested., we chose to sequence genomic DNA of the non-adherent bacterial fraction as the adherent fraction is less pure since it includes bacteria that adhere to the biofilm after 24 hours, while the non-adherent fraction is purely non-adherent to the fibrin matrix or the biofilm itself. After a first infection of the fibrin matrix for 24 hours, the non-adherent bacteria from two independent experiments were sequenced but showed no significant differences in the mutagenized genes that were found in this output pool compared to the input pool, which represented the complete transposon library (**Figure 1C**). In **Figure 1D**, this comparison was executed again two times with the output pool after the second infection. For this second incubation, the non-adherent bacteria harvested after the first incubation round were used to infect a new fibrin matrix (**Figure 1A**). This procedure resulted in a selection of genes with a significant difference in read counts, and therefore mutant frequencies, in the pool of the non-adherent bacteria. This result indicates that we can distinguish between genes that play a role in the adherence to fibrin and biofilm formation and those that do not. In **Figure 1E**, genes with a significant log2FC>2 of clusters of orthologous genes (COGs) G, M, V and T, which encode carbohydrate transport and metabolism, cell wall biogenesis, defense mechanisms and signal transduction mechanisms, are shown to be enriched in the second selection (right column) compared to the first selection (left column), which is seen from the increase in log2FC values. In this subset, we find genes like rfbA-D and galE, which encode factors that play a role in the biosynthesis of extracellular polysaccharides. To analyze the biological functions and pathways of the encoded factors that are involved in biofilm formation, we applied further pathway analysis.

### 3.2 Metabolic pathways and signaling pathways like quorum sensing and HIF-1 are involved in biofilm formation on a fibrin matrix under flow conditions

**Figure 2:**
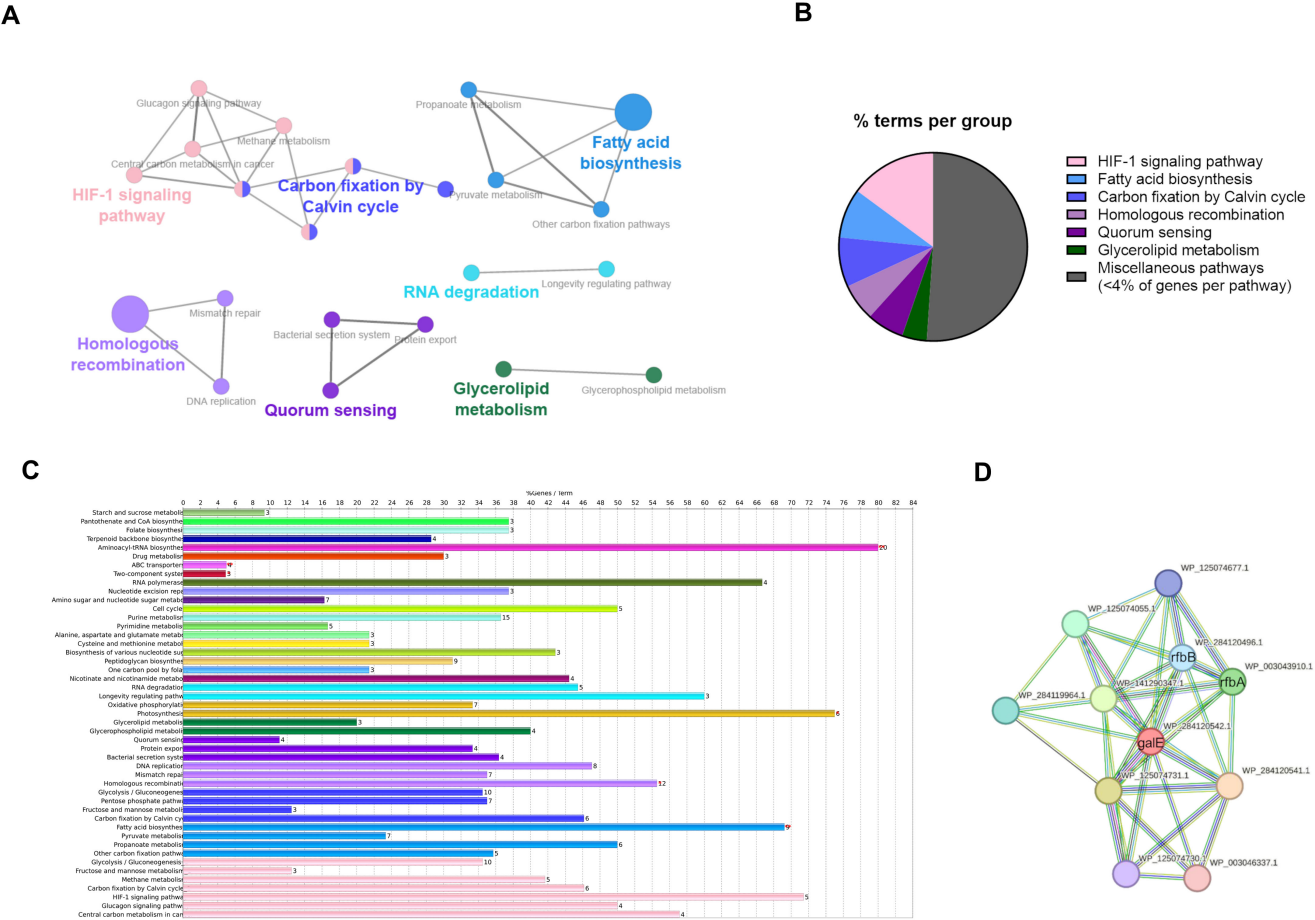
**(A)** KEGG pathway enrichment of genes with log2FC>2 and q<0.01 after second selection step, including only pathways that show interactions between them. **(B)** Pie chart showing the percentage of the genes in a certain KEGG pathway found in the enrichmentanalysis. A total of 22 pathways that had percentages under 4% were grouped under miscellaneous. **(C)** Bar chart showing percentage of the total genes of each separate KEGG pathway involved in biofilm formation of (S) canis. **(D)** STRING protein-protein interaction network highlighting the interactions of genes of the rfb operon and galE.

KEGG enrichment analysis of biofilm-associated genes revealed significant representation of pathways involved in carbohydrate metabolism, cell wall biosynthesis, and QS in the TraDIS output pool after selecting for biofilm formation on a fibrin matrix (**Figures 2A–D**). A list of enriched genes is added as **Supplementary Table 1**. In **Figure 2A**, results of KEGG pathway enrichment analysis demonstrate how the involved genes are connected. **Figure 2B** illustrates how many genes of the sequenced TraDIS output pool were assigned to a certain KEGG pathway. In addition to an remarkable enrichment of genes involved in HIF-1 signaling (pink), fatty acid biosynthesis (light blue), carbon fixation (blue), homologous recombination (light violet), Quorum sensing (violet), and glycerol lipid metabolism (green). Over 50% of the genes were found in a total of 22 other pathways, with less than 4% of involved genes in each respective pathway. These were labelled as miscellaneous (dark grey) and not considered in our further analysis. **Figure 2C** presents a list of genes from the TraDIS output pool as a percentage of the total amount of genes in that specific KEGG pathway using the same color coding as **Figures 2A, B**. This highlights the importance of many metabolic pathways and of the HIF-1 signaling pathway in streptococcal attachment to fibrin matrices and reveals a large percentage of genes functionally related to biofilm formation. Protein-protein interaction mapping using the IMT49926 proteome in the STRING database identified a tightly connected network of the RfbA-D-encoding operon and *galE* (**Figure 2C**), linking gene products that are involved in biosynthesis of extracellular polysaccharides. Together, these results suggest that biofilm development in *S. canis* involves coordination between metabolic activity, QS, HIF-1 signaling and homologous recombination.

### 3.3 Carvacrol can inhibit biofilm formation of *S. canis* by suppressing LuxS-dependent quorum sensing

**Figure 3:**
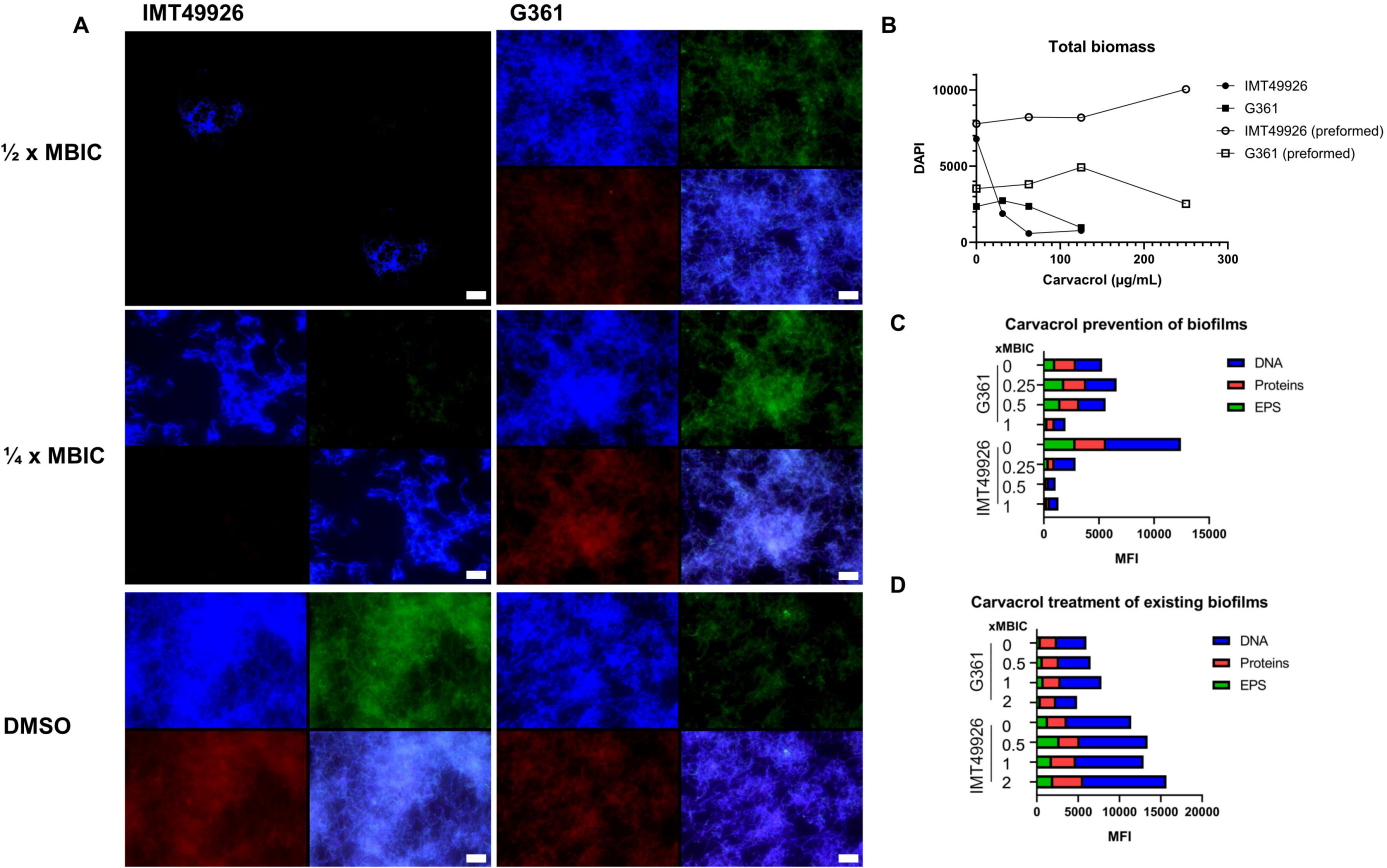
**(A)** Immunofluorescent microscopy images of (S) canis IMT49926 and G361 biofilms treated with carvacrol at ½ × and ¼ × the minimum biofilm inhibitory concentration (MBIC) or vehicle control (DMSO). Biofilm components were stained to visualize DNA (blue, DAPI), exopolysaccharides (green, wheat germ agglutin (WGA-AF488)), and proteins (red, FilmTracer Ruby Biofilm Matrix Stain). Carvacrol reduced the overall biofilm density compared to DMSO-treated controls, especially in the IMT49926 strain. Images shown are representative maximum-intensity projections, composite overlays are presented for visualization only. Scale bars represent 5 µm. **(B)** Quantification of mean fluorescence intensity (MFI) of the DAPI channel as a measure of total biofilm biomass following exposure to increasing concentrations of carvacrol, showing a dose-dependent inhibition for both isolates but no reduction for both their preformed biofilms. **(C)** MFI quantification of DNA, protein, and EPS components in biofilms formed in the presence of carvacrol (biofilm prevention assay). **(D)** MFI quantification of DNA, protein, and EPS components in preformed biofilms treated with carvacrol (biofilm disruption assay). MFIs were quantified independently for each fluorescent channel following background subtraction using identical acquisition settings across conditions and were not summed across channels. Apparent intensity differences in representative images reflect display scaling; raw fluorescence values used for quantitative analysis were not saturated.

To assess the role of LuxS-dependent QS in *S. canis* biofilm formation, we compared the wild-type strain IMT49926 and *S. canis* strain G361 in the presence of carvacrol, a substance that has been shown to inhibit and eradicate *S. pyogenes* biofilms via downregulation of *luxS* and reducing cell surface hydrophobicity (Wijesundara et al., 2022). Since IMT49926 forms a biofilm that is about twice the total biomass of the G361 biofilm (**Figure 3B**) measured by MFI of DAPI, we expect that carvacrol would have an increased effect on IMT49926, as decreased AI-2 levels are caused by lower expression of *luxS*, leading to reduced biofilm formation. After confirming that the minimum biofilm inhibitory concentration (MBIC) for both *S. canis* strains was the same as the published MBIC for *S. pyogenes*, 125 µg/mL, biofilms were grown under sub-MBIC concentrations after which matrix components were stained. Immunofluorescent microscopy revealed that carvacrol treatment substantially disrupted biofilm architecture and reduced extracellular matrix accumulation in the IMT49926 strain in a concentration-dependent manner (**Figures 3A–C**). In contrast, the G361 formed thinner, less structured biofilms but was significantly less sensitive to carvacrol, maintaining a greater proportion of biomass and matrix components (**Figures 3A–C**). As shown in **Figures 3B–D**, treatment of preformed biofilms did not result in reduction of biofilm mass, indicating that quorum-sensing blockade primarily affects the early stages of biofilm development and the eradication of mature biofilms that was shown for *S. pyogenes*, could not be repeated for *S. canis*. Together, these findings suggests that carvacrol inhibits *S. canis* biofilm formation by targeting LuxS-dependent quorum sensing, which would be in line with formerly cited published observations.

### 3.4 The serum opacification factor of *S. canis* (ScSOF) contributes to fibronectin-mediated biofilm development and extracellular matrix composition

**Figure 4:**
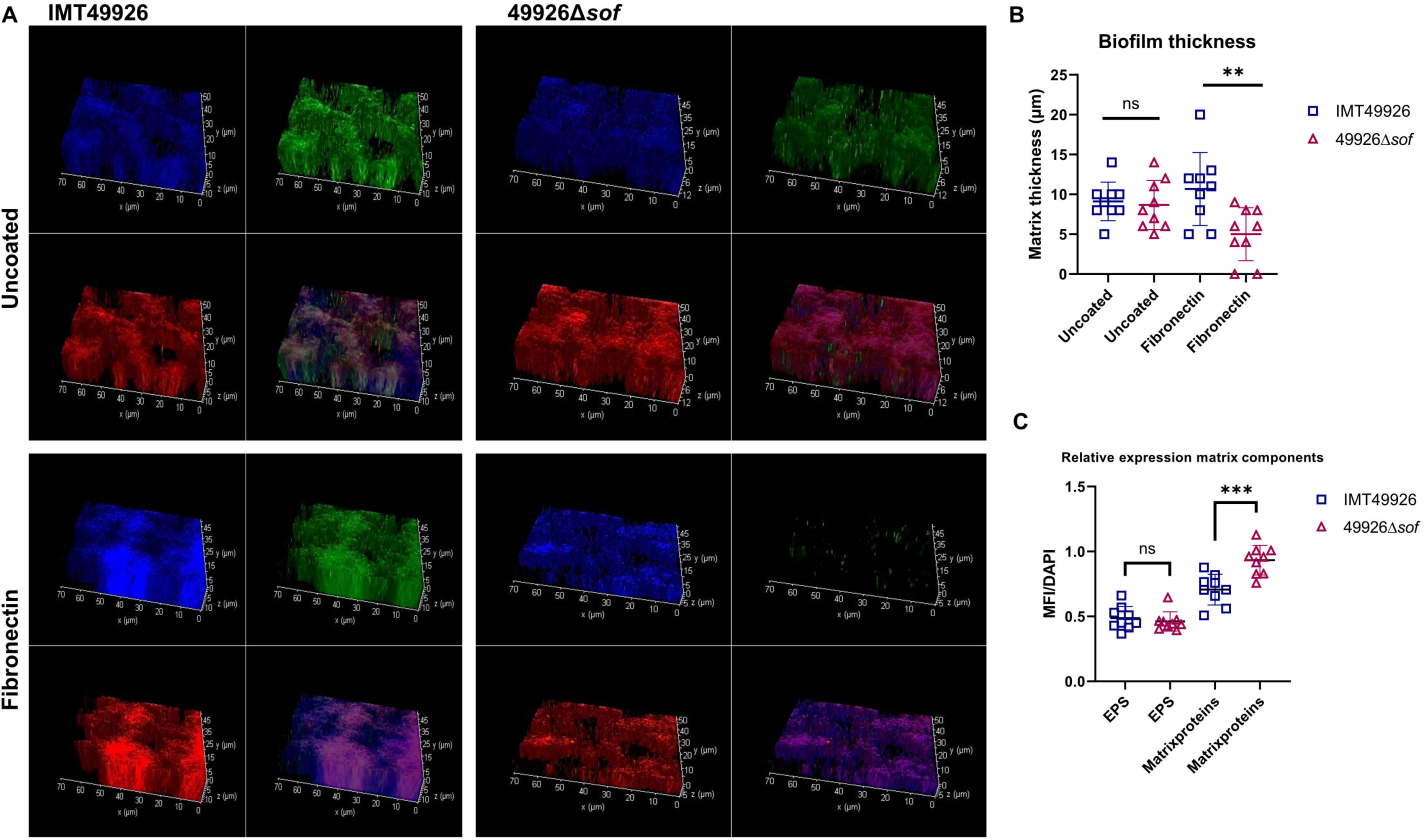
**(A)** Representative 3D immunofluorescent microscopy reconstructions of biofilms formed by (S) canis IMT49926 (wild type, WT) and its isogenic Δs of mutant on uncoated and fibronectin-coated surfaces. Biofilm components were stained for DNA (blue, DAPI), exopolysaccharides (green, WGA-AF488), and proteins (red, FilmTracer Ruby Biofilm Matrix Stain). Composite images are shown for visualization of biofilm architecture; individual fluorescent channels were acquired sequentially and quantified independently. WT biofilms formed on fibronectin exhibited increased structural density and matrix organization compared to Δsof biofilms. **(B)** Quantification of biofilm matrix thickness. WT (S) canis biofilms were significantly thicker on fibronectin-coated surfaces compared to uncoated, whereas Δsof biofilms showed reduced thickness overall. Data represents mean ± SD from three independent experiments; p values determined by one-way ANOVA with *post hoc* comparisons (ns = not significant, **p < 0.01). **(C)** Relative mean fluorescence intensity (MFI) ratios of exopolysaccharides (EPS) and proteins to DNA, comparing WT and Δsof biofilms grown for 72 hours on uncoated surfaces. MFIs were calculated per channel without summation to account for potential spatial overlap between fluorophores. Sof deletion increased matrix protein abundance, but not EPS expression Data represent mean ± SD from three independent experiments; p values determined by one-way ANOVA with *post hoc* comparisons (ns, not significant, ***p < 0.001).

In our previous study, we found the serum opacification factor of *S. canis* (ScSOF) to be involved in adherence and invasion of endothelial and epithelial cells (Katsburg et al., 2025). As fibronectin is abundant in host tissues, such as damaged heart valves, and ScSOF is a fibronectin-binding protein, we wanted to investigate the function of ScSOF in creating biofilms on a fibronectin surface as well as a neutral surface. Immunofluorescent microscopy showed that the fibronectin-binding serum opacification factor of *S. canis* does not interfere with biofilm formation on a non-biological surface (**Figures 4A, B**). However, if the surface is coated with fibronectin, a significant reduction in biofilm is shown for the targeted *sof* knockout mutant, even after 72 hours of incubation. Interestingly, the *sof* knockout mutant showed altered matrix composition on uncoated surfaces, with comparable expression of extracellular polysaccharides (EPS), but increased amount of extracellular matrix proteins, as shown in **Figure 4C**. Taken together, this shows how initial adherence to the host matrix proteins is a crucial step in biofilm formation. It also shows that this surface protein of *S. canis* might have a different function besides fibronectin-binding, as a lack of it increases the amount of total matrix proteins in the biofilm without increasing the total biomass, potentially through a role in regulation of the extracellular matrix expression.

## 4 Discussion

Biofilm formation is a critical virulence trait in *S. canis* during infective endocarditis and is one of the main challenges for antibiotic treatment of heart valve infections. Bacterial biofilms are characterized as three-dimensional structures, mainly composed of the exopolysaccharide matrix, which is produced and secreted by the bacteria and which is directly followed by bacterial adhesion leading to colonization. The bacterial exopolysaccharide matrix is known to mediate bacterial attachment to surfaces, which enables the use of biofilm-mediated adherence as functional read out for biofilm formation. Implementation of defined flow environment is a well-known technique to discriminate between weak and strong biofilm formers as had been demonstrated for *Streptococcus agalactiae* by Pieranski et al., 2021. By combining a genome-wide TraDIS screen with biofilm assays under physiologically relevant flow conditions on a fibrin matrix, we identified multiple gene networks and regulatory pathways that promote biofilm establishment. Our results highlight a complex interplay between carbohydrate metabolism, extracellular polysaccharide biosynthesis, quorum sensing, and signaling pathways, emphasizing that *S. canis* biofilm formation is a multifactorial process shaped by metabolic and environmental cues.

The screen for bacterial factors mediating biofilm attachment was achieved by a negative selection process, which filters bacterial transposon mutants with functional loss of adherence due to single transposon insertions by a negative selection approach. This approach required two subsequent steps of screening which resulted in an enrichment of mutations found in genes related to carbohydrate metabolism (COG: G), cell wall biogenesis (COG: M), and signal transduction mechanisms (COG: T) among the transposon mutants that were unable to partake in biofilm formation. As we initially expected, the identification of the *rfb* operon (*rfbA–D*) and *galE* supports a central role for extracellular polysaccharide biosynthesis in *S. canis* biofilm architecture. The *rfb* operon is crucial for dTDP-rhamnose biosynthesis, an important precursor of cell wall polysaccharides and extracellular polysaccharides as shown in *Lactococcus lactis* (Boels et al., 2004). Rhamnose-containing cell wall polysaccharides play a crucial role in the cell wall architecture and pathogenesis of streptococci and enterococci (Mistou et al., 2016). These polysaccharide components likely play an important role in the structural stability of the biofilm matrix, especially under the dynamic conditions of generating cardiac vegetations (Limoli et al., 2015; Lerche et al., 2021a).

It has to be noted that in pooled transposon libraries, extracellular polymers and other matrix components are shared within the developing biofilm, allowing mutants with impaired biofilm-associated functions to persist in the population without immediately displaying a strong fitness defect. The fact that two subsequent selection rounds for gene enrichment were required to achieve significant results might point to the phenomenon of “biofilm cheating,” in which mutants that are defective in the production of extracellular matrix components or adhesion factors can still benefit from the public goods produced by neighbouring cells within the population (Popat et al., 2012). As a result, differences in mutant abundance may remain subtle after a single round of selection. To increase the sensitivity of the screen, we therefore performed a second round of selection using the non-adherent fraction from the first round. This iterative enrichment approach amplifies subtle fitness differences and is commonly used in pooled transposon sequencing strategies to detect genes with moderate phenotypic contributions. After the second selection step, significant enrichment of mutants in genes associated with carbohydrate metabolism, cell wall biogenesis, and signalling pathways became apparent. These results suggest that repeated selection is necessary to overcome the buffering effects of communal biofilm traits and to reveal genes contributing to biofilm formation under physiologically relevant flow conditions. KEGG pathway enrichment further identified QS among the overrepresented pathways. Especially the LuxS/AI-2 quorum sensing system appears to play an important role in *S. canis* biofilm formation. The clinical endocarditis strain IMT49926 formed robust biofilms that were sensitive to inhibition by carvacrol, a proposed LuxS/AI-2 signaling inhibitor in S*. pyogenes*. *S. pyogenes* produces a nearly identical *luxS* gene product compared to *S. canis* (99.9% translated protein sequence similarity). In contrast, strain G361 produced thinner biofilms and showed reduced responsiveness to carvacrol, which might indicate that increased AI-2 quorum sensing and biofilm formation would lead to increased sensitivity to carvacrol. Nevertheless, it has to be taken into account that the detected differences in amount of biofilm between *S. canis* IMT49926 and G361 might also reflect differences in expression of surface adhesins and the growth behavior within biofilm, although bacterial growth in liquid media was not significantly different. Carvacrol was also able to eliminate a preformed mature biofilm of *S. pyogenes* by reducing cell surface hydrophobicity (Wijesundara et al., 2022). In contrast, a full elimination of preformed biofilms was not achieved for both *S. canis* strains. This pattern might indicate that LuxS-mediated signaling predominantly promotes early biofilm development and matrix development, and that carvacrol’s antibiofilm effect is primarily due to interference with this pathway. Comparable findings have been reported in *S. mutans* and *S. suis*, where LuxS disruption alters biofilm architecture and extracellular polymer production (Merritt et al., 2003; Yoshida et al., 2005). Overall, the results obtained from biofilm formation in presence of the LuxS-targeting inhibitor carvacrol provide initial evidence supporting a potential role of quorum sensing in biofilm formation of *S. canis* although further experimental approaches involving a luxS knockout and AI-2 quantifications are needed to elucidate the underlaying mechanistic details in a follow-up project.

While QS is already known to play an important role in infective endocarditis, HIF-1 signaling is particularly interesting. HIF-1-related genes have been linked to bacterial adaptation under oxygen-limited and stress conditions within host tissues, suggesting that *S. canis* may exploit metabolic reprogramming and signaling cross-talk to thrive in the hypoxic microenvironment of an infected heart valve (Werth et al., 2010; Devraj et al., 2017; Thompson et al., 2017). HIF-1 is believed to upregulate glycolytic genes to boost immunity and reduce bacterial invasion. However, the bacteria will also upregulate glycolysis pathways to adapt to the oxygen-poor environment, therefore potentially creating competition for glucose with the host cells in the biofilm environment (Kedia-Mehta et al., 2019; Mirghani et al., 2022). Pharmacological stabilization of HIF-1 with prolyl hydroxylase inhibitors such as AKB-4924 could represent an interesting new treatment strategy for *S. canis* endocarditis. HIF-1 activation could mitigate bacterial persistence and improve antibiotic efficacy, by enhancing endothelial resistance to invasion and boosting host innate defenses (Beasley et al., 2013).

The enrichment of HIF-1 pathway associated genes points to a further important correlation. The list of enriched genes assigned for the HIF-1-pathway include genes encoding key enzymes of the glycolysis such as enolase (*eno*), glyceraldehyde-3-phosphate dehyrogenase (*gap*), and 3-phosphoglycerate kinase (*pgk*). For most of these glycolytic enzymes, bacterial surface displayed moonlighting activity has already been demonstrated for *S. canis* and other Streptococcal species such as *Streptococcus pneumoniae*, *Streptococcus suis*, *Streptococcus thermophilu*s, *Streptococcus iniae*, and *Streptococcus pyogenes* (Fulde et al., 2013; Bergmann et al., 2001; Zhao et al., 2024; Mu et al., 2020; Wang et al., 2015; Ayinuola et al., 2022). In this respect, moonlighting activity is defined as an additional protein function, which in many cases covers specific virulence traits such as adhesion activities. For example, in former own studies we identified the enolase of *S. canis* and also the enolase of *Streptococcus pneumoniae* as surface-exposed binding protein for host-derived plasminogen, which promotes proteolytical fibrin dissolution and bacterial dissemination (Fulde et al., 2013; Bergmann et al., 2001). Even in the absence of plasminogen, a direct contribution of the enolase of *Streptococcus suis* to bacterial translocation across the blood brain barrier has been demonstrated, which indicates that the moonlighting activity of surface exposed metabolic proteins might directly or at least indirectly contribute to bacterial attachment to fibrin surfaces (Zhao et al., 2024). Likewise, own former studies identified the surface exposed 3-phosphoglycerate kinase as plasminogen binding protein and tissue activator of *S. pneumoniae* (Fulde et al., 2013). A direct contribution to bacterial adherence to host cells was already demonstrated for the SDH, the streptococcal glyceraldehyde-3-phosphodehydrogenase of *S. pyogenes* (Jin et al., 2005). Even if no surface display had been demonstrated for glyceraldehyde-3-phosphodehydrogenase of *S. canis* yet, GAP belongs to the family of bacterial moonlighting proteins, thereby sharing a great likelihood for similar functions regarding both, i) metabolic activity for biofilm production, and ii) bridging mediator for attachment to fibrin surfaces, respectively.

It has to be noted that surface appendages such as pili or flagellae, which serve as potent surface adhesins do not belong to the attachment protein repertoire of *S. canis.* That aside, in former studies, we already identified the M protein of *S. canis* as surface-displayed adhesin mediating a cooperative plasminogen recruitment (Fulde et al., 2013; Fulde et al., 2011). Most interestingly, the M protein was also found responsible for a hemophilic protein re-association, thereby promoting bacterial aggregation in an immune protective manner (Fulde et al., 2013). This hemophilic self-aggregation might also be of relevance for providing bacterial stabilization within a growing biofilm matrix. Moreover, a study of Fukushima and colleges reported a direct correlation between the expression of certain *scm* variants of *S. canis* with the ability to produce biofilm (Fukushima et al., 2022). Therefore, the successful enrichment of the *scm* gene in the transposon library screening analyses can be considered as a positive validation of the used complex techniques. In addition to the *scm* gene enrichment, the screening results identified another gene encoding a surface anchored protein, which was identified as fibronectin-binding serum opacity factor (ScSOF) of *S. canis*. The ScSOF plays a role in initial adherence to the host matrix protein fibronectin. While *sof* deletion did not affect biofilm development on uncoated surfaces, the Δ*sof* mutant displayed significantly reduced biofilm thickness on fibronectin-coated surfaces, demonstrating that SOF facilitates host matrix-dependent adhesion and biofilm growth. This suggests that the initial adherence step to the host matrix proteins is a crucial step in the development of infective endocarditis, which is characterized by a mature biofilm on the endocardium (Keynan et al., 2013). Interestingly, the absence of ScSOF also altered the composition of the biofilm matrix: the mutant produced more extracellular matrix proteins. These findings suggest that ScSOF not only mediates surface attachment but may also modulate regulatory pathways controlling matrix biosynthesis. This dual role mirrors findings in other streptococci, where SOF-like and M-like proteins coordinate adhesion and immune evasion, thereby influencing both colonization and persistence (Oehmcke et al., 2010; Fulde et al., 2013; Zhu et al., 2017). In order to analyse the impact of SOF in biofilm-mediated diseases such as an infective endocarditis more to detail, the use of sophisticated cell culture infection models under defined microfluidic also including host immune components would provide the possibility to gain more insight into the underlaying bacterial pathomechanisms. Moreover, in former studies we already demonstrated a significantly accelerated fibrinolysis due to *S. canis* surface adhesins (2011; 2013). Since bacterial vegetations at infected heart valves are characterized by an inflammation-triggered enhanced coagulation cascade, surface-recruited proteolytic activity of host-derived plasmin might have an effect on biofilm stability and biofilm dissolution. In this respect, further studies will unravel whether M- protein and SOF of *S. canis* act synergistically to stabilize the biofilm, mediate attachment to fibrin surfaces, and finally recruite host-derived proteolytic activity to mediate bacterial dissemination into deeper tissue sides. These results might indicate that biofilm-mediated attachment of *S. canis* mostly depends on metabolic pathways rather than on single virulence traits.

Together, our results support a model in which *S. canis* biofilm formation during infective endocarditis depends on (i) metabolic pathways supporting extracellular polysaccharide synthesis, (ii) LuxS-dependent quorum sensing for biofilm regulation and maturation, and (iii) adhesin-mediated binding to host matrix proteins such as fibronectin and fibrin. The identification of host-matrix-responsive pathways and quorum-sensing-regulated biofilm mechanisms underscore potential therapeutic targets for disrupting formation and potential elimination of *S. canis* biofilms, particularly in endocardial infections where fibrin-rich vegetations protect bacteria from antibiotic clearance (Keynan et al., 2013; Lerche et al., 2021a).

This study provides the first integrated analysis of biofilm-associated genes and pathways in *S. canis* under flow conditions that mimic the blood-flow mediated shear stress in the infective endocarditis environment. The discovery of links between carbohydrate metabolism, quorum sensing, and surface adhesins expands our understanding of *S. canis* pathophysiology. Future work should focus on expansion of the dataset using metabolomics and proteomics to further elucidate the crucial metabolic pathways for the formation of biofilms in infective endocarditis.

## Data Availability

Sequencing data generated and analyzed in this study have been deposited in the NCBI BioProject repository and are publicly available under BioProject accession: PRJNA945807, BioSample accessions SAMN53665527; SAMN54325065-SAMN54325068.
